# Role of hypoxia-mediated pyroptosis in the development of extending knee joint contracture in rats

**DOI:** 10.1186/s40001-024-01890-9

**Published:** 2024-05-27

**Authors:** Quan-Bing Zhang, Lei Huo, Mian Li, Rui Zhang, Ting Zhou, Feng Wang, Yun Zhou

**Affiliations:** 1 Department of Rehabilitation Medicine, The Second Affiliated Hospital of Anhui Medical University, No.678 Furong Road, Economic and Technological Development Zone, Hefei, 230601 Anhui China; 2 Hefei Institute of Physical Sciences, Chinese Academy of Sciences, Hefei, 230031 Anhui China

**Keywords:** Joint contracture, Joint capsule fibrosis, HIF-1α/NLRP3 signaling pathway, Pyroptosis, TGF-β1/Smad3 signaling pathway

## Abstract

Joint contracture is one of the common diseases clinically, and joint capsule fibrosis is considered to be one of the most important pathological changes of joint contracture. However, the underlying mechanism of joint capsule fibrosis is still controversial. The present study aims to establish an animal model of knee extending joint contracture in rats, and to investigate the role of hypoxia-mediated pyroptosis in the progression of joint contracture using this animal model. 36 male SD rats were selected, 6 of which were not immobilized and were used as control group, while 30 rats were divided into I-1 group (immobilized for 1 week following 7 weeks of free movement), I-2 group (immobilized for 2 weeks following 6 weeks of free movement), I-4 group (immobilized for 4 weeks following 4 weeks of free movement), I-6 group (immobilized for 6 weeks following 2 weeks of free movement) and I-8 group (immobilized for 8 weeks) according to different immobilizing time. The progression of joint contracture was assessed by the measurement of knee joint range of motion, collagen deposition in joint capsule was examined with Masson staining, protein expression levels of HIF-1α, NLRP3, Caspase-1, GSDMD-N, TGF-β1, α-SMA and p-Smad3 in joint capsule were assessed using western blotting, and the morphological changes of fibroblasts were observed by transmission electron microscopy. The degree of total and arthrogenic contracture progressed from the first week and lasted until the first eight weeks after immobilization. The degree of total and arthrogenic contracture progressed rapidly in the first four weeks after immobilization and then progressed slowly. Masson staining indicated that collagen deposition in joint capsule gradually increased in the first 8 weeks following immobilization. Western blotting analysis showed that the protein levels of HIF-1α continued to increase during the first 8 weeks of immobilization, and the protein levels of pyroptosis-related proteins NLRP3, Caspase-1, GSDMD-N continued to increase in the first 4 weeks after immobilization and then decreased. The protein levels of fibrosis-related proteins TGF-β1, p-Smad3 and α-SMA continued to increase in the first 8 weeks after immobilization. Transmission electron microscopy showed that 4 weeks of immobilization induced cell membrane rupture and cell contents overflow, which further indicated the activation of pyroptosis. Knee extending joint contracture animal model can be established by external immobilization orthosis in rats, and the activation of hypoxia-mediated pyroptosis may play a stimulating role in the process of joint capsule fibrosis and joint contracture.

## Introduction

Joint contracture is one of the common diseases in department of rehabilitation medicine, which seriously affects patients' activities of daily living [1, 2]. With the development of modern transportation and construction industry, the number of patients with lower limb trauma is increased. To promote tissue healing, joint immobilization is usually employed. However, long-term or inappropriate joint immobilization may easily lead to joint contracture, resulting in the reduction of patients' quality of life [3, 4]. Consequently, understanding of the mechanism on joint contracture is of great importance to the treatment.

Joint capsule fibrosis is one of the most important pathological changes after joint immobilization, and is also the key reason for the occurrence and development of joint contracture [5]. Previous studies have shown that the activation of TGF-β/Smad signaling pathway is the most direct and important signaling pathway causing joint capsule fibrosis [2, 5, 6]. Activation of this pathway involves multiple cytokine interactions. Among the cytokines involved in TGF-β/Smad signaling pathway, transforming growth factor β1 (TGF-β1) is known to be a main inducer of the initiation and maintenance of fibrosis, which can promote cell differentiation, proliferation and extracellular matrix production, cause collagen deposition and increase expression of α-SMA, a biomarker for myofibroblast differentiation [7–9]. Smads pathway is the most important pathway in TGF-β1 signal transduction. Smad2 and Smad3 are the first signal molecules transmitted by TGF-β1 signal, among which Smad3 is more closely related to fibrosis [10–12]. TGF-β1/Smad3 is a well-studied signaling pathway for joint capsule fibrosis and joint contracture induced by joint immobilization. However, the exact regulatory mechanism of TGF-β1/Smad3 signaling pathway in immobilization-induced joint capsule fibrosis remains unclear.

Pyroptosis is a type of programmed cell necrosis mediated by GSDMD [13]. Caspase-1, which mediates classical pathway of pyroptosis, belongs to the caspase subfamily and play key roles in immune response-related signaling [14]. GSDMD, which is identified as a substrate of caspase-1, is primarily expressed in immune cells and shows unique structural characteristics of a perforating protein [15]. Inflammasome such as NLRP3 senses a variety of exogenous or endogenous danger signals, and activates caspase-1 through the coupling molecule ASC, thereby cutting GSDMD, dissociating the N-terminal from the C-terminal. Self-inhibition is subsequently broken, and honeycomb channels are formed through oligomers on cytomembrane following interaction of positive and negative charges. Then the cell suffered from swelling, osmotic dissolution, rupture and death, which indicated the induction of pyroptosis [16]. Once pyroptosis is activated, intracellular contents is released and a series of subsequent physiological processes can be induced [17, 18]. Previous studies have shown that pyroptosis can activate TGF-β/Smad pathway in liver, kidney, lung and many other tissues, and then lead to tissue fibrosis [19–21]. Nevertheless, studies on the role of pyroptosis in immobilization-induced joint capsule fibrosis are still unclear.

Previous studies have shown that joint contracture can be caused by hypoxia of joint capsule when knee joint is fixed in straight position in rat, which may be an important initiating factor of joint contracture [22]. Adaptation to hypoxia environment is mainly regulated through hypoxia-inducible factor 1 (HIF-1α), which is an environmental sensor orchestrating the adaptation to environmental changes [23]. Under hypoxia environment, HIF-1α subunit is stabilized, allowing translocation to the nucleus, heterodimerization, and activation of gene pathways that minimize oxygen consumption, reduce reactive oxygen species (ROS), and restore oxygen delivery [24]. Previous studies have indicated that HIF-1α can promote the activation of NLRP3 inflammasome, facilitate the activation of pyroptosis [25, 26]. Zhang L et al. [27] reported that hypoxia environment can induce NRLP3 activation in synovial tissues of knee joint with osteoarthritis, and then stimulate pyroptosis, activate TGF-β/Smad signaling pathway and promote tissue fibrosis.

Knee joint contracture is a very difficult disease clinically. Joint capsule fibrosis is the most important pathological change causing joint contracture, and hypoxia is an initiating factor causing joint capsule fibrosis. Previous studies have suggested that hypoxia can induce the activation of pyroptosis in the progression of various other diseases, but the role of pyroptosis in immobilization-induced joint capsule fibrosis and joint contracture remains unclear. Based on this, the present study aims to establish an animal model of knee joint extension contracture in rats, and to explore the role of pyroptosis mediated by hypoxia in the pathogenesis of knee joint contracture induced by immobilization in rats, so as to provide theoretical basis for the timely and effective treatment of knee joint contracture in the future.

## Materials and methods

### Animals and experimental materials

**Fig. 1:**
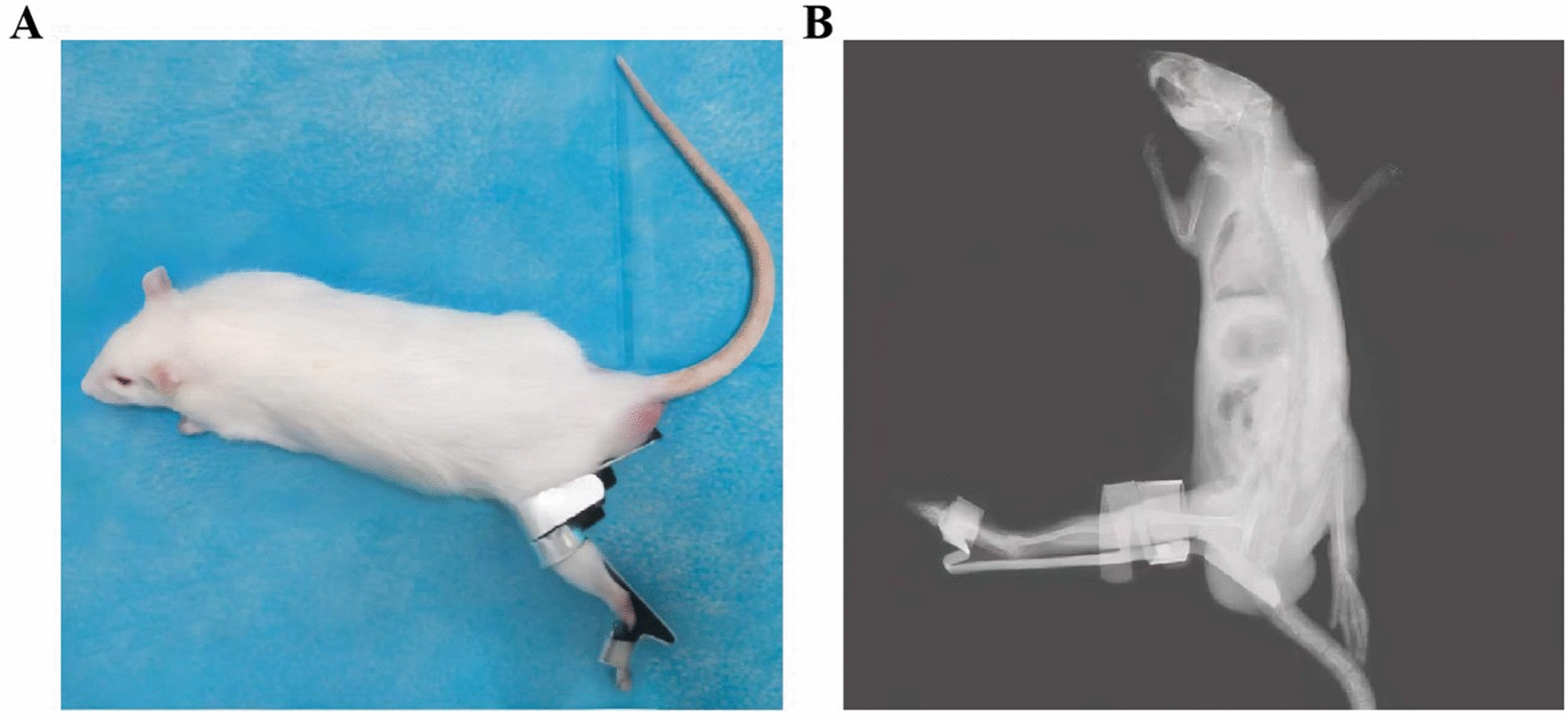
Animal model of rat knee joint contracture. **A** Homemade aluminum plate was used to immobilize the knee of rat at extension from the groin to the proximal toes. **B** X-ray observation was used to confirm the knee extending immobilization

The present study was approved by the Institutional Animal Care and Use Committee of Anhui Medical University (NO. LLSC20221126). 36 male SD rats were purchased from the Experimental Animal Center of Anhui Medical University (age, 3 months; weight, 250-300 g). The experimental rats were produced by Jinan Pengyue Experimental Animal Breeding Co., Ltd. The animals were closed colonies of SD rats with good health condition and no diseases of bone and joint system at the time of purchase. 30 rats were randomly divided into 5 experimental groups with 6 rats in each group: I-1 group (immobilized for 1 week following 7 weeks of free movement), I-2 group (immobilized for 2 weeks following 6 weeks of free movement), I-4 group (immobilized for 4 weeks following 4 weeks of free movement), I-6 group (immobilized for 6 weeks following 2 weeks of free movement), I-8 group (immobilized for 8 weeks). Molded aluminum splint was used to immobilize the knee of rat at extension from the groin to the proximal toes to establish the animal model of joint contracture (Fig. 1, utility model patent number: ZL202120470158.0). The rats were housed at 22–24 ℃ with a 12-h light/dark cycle and were allowed free access to food and water. X-ray observation was used to confirm the knee extending immobilization in our study. A control group of six rats underwent 8 weeks of free movement.

### Range of motion measurements

**Fig. 2:**
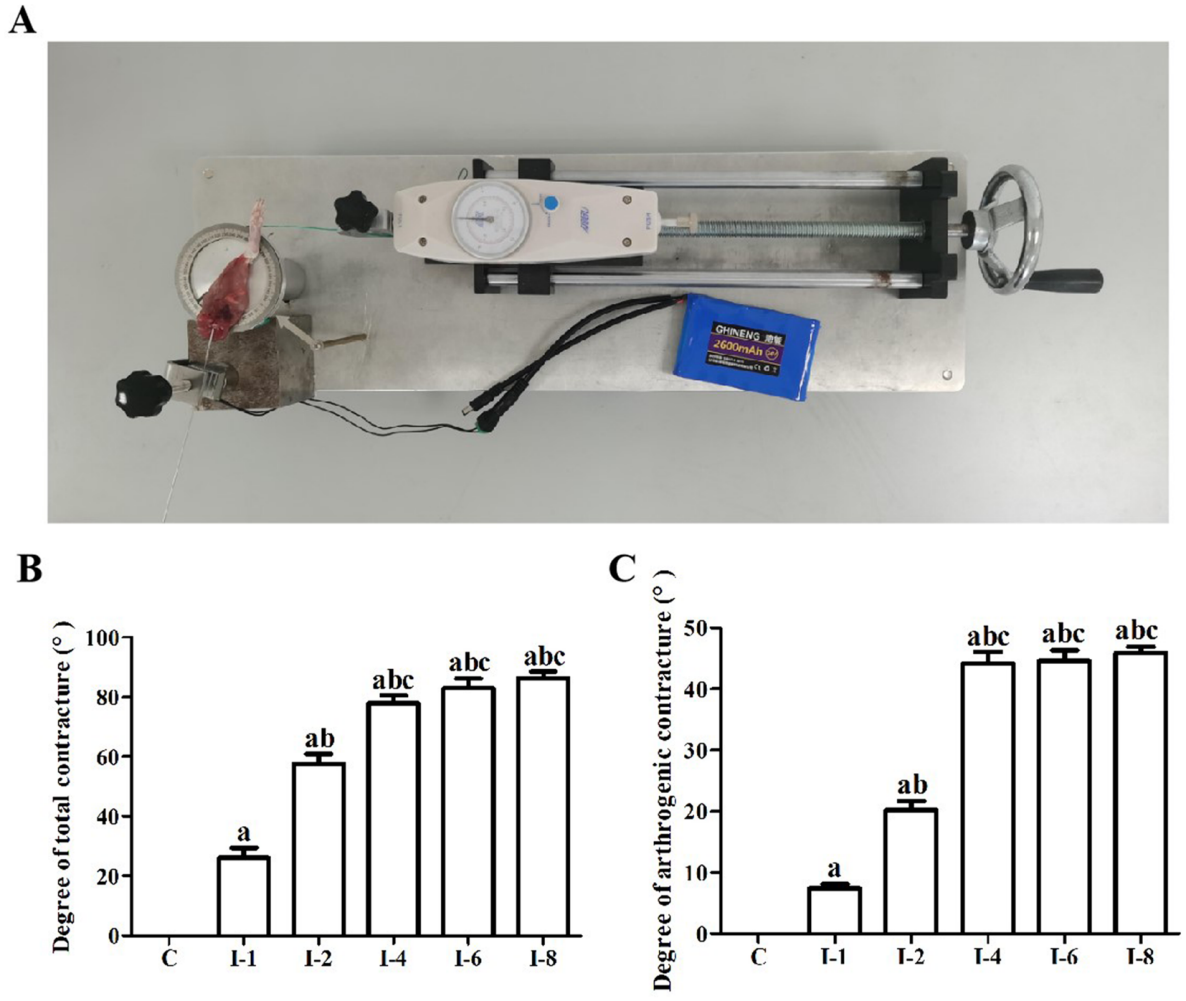
Immobilization induced total and arthrogenic contracture in rats. **A** A mechanical goniometer used for joint range of motion measurement. **B** Quantitative analysis of the degree of total contracture. **C** Quantitative analysis of the degree of arthrogenic contracture. C, rats that did not undergo immobilization; I-1, rats that underwent 1 week of immobilization; I-2, rats that underwent 2 weeks of immobilization; I-4, rats that underwent 4 weeks of immobilization; I-6, rats that underwent 6 weeks of immobilization; I-8, rats that underwent 8 weeks of immobilization

The rats were euthanized via an overdose of sodium pentobarbital and the joint ranges of motion were measured with a mechanical goniometer (Fig. 2A, utility model patent number: ZL202120996643.1). The specific measurement principles and methods are as follows:

1. Ergometer connection: A precise ergometer was fixed on a pedestal which was placed on the slideway equipment. A string was used to connect the groove of the disc and the ergometer.
2. Calibration of the mechanical goniometer: The mechanical goniometer was calibrated before application. The detailed calibration information is as follows: the force measured on the ergometer was set as Fa. The reaction force of the string that connected to the groove of the disc and the ergometer was set as Fb. The resistance generated by the self-damping of the measuring instrument was set as Fc, and Fc was the measurement error (including the static friction force of the disc, the resistance generated by the bearing damping of the disc axis, and the resistance generated by the self-damping of the static pulley). The force that drives the knee joint of the rat to an certain angle was set as Fd. According to laws of Newtonian mechanics, Fa = Fb = Fc + Fd. When no rats were tied up and the device was unloaded, Fd = 0 and Fa = Fb = Fc. Then we set zero calibration to the dynamometer at this point. The above operation eliminates the error of detection, and the reading on the ergometer (Fa) only reflects the force that drives the knee joint of the rat to a certain angle (Fd).
3. Rat hip dislocation: The rats were killed with an overdose of sodium pentobarbital. The immobilized hind limbs of the rats were disarticulated at the hip joint.
4. Lower limb fixation: A Kirschner wire was inserted into the femur and the Kirschner wire was fixed with a removable metal clip connected with an electromagnet. Disposable plastic cable ties were used to fix the proximal and distal parts of the tibia on the disc.
5. Measuring operation: The disc can be twisted after the driving wheel was turned, and the tibia was indirectly twirled with the femur being static.
6. Torque calculation: The force adopted can be seen on the ergometer and the joint range of motion can be measured according to the scale on the disc. The torque which was calculated via multiplying the force by the constant radius of the disc depended linearly on the force applied.
7. Torque selection: Prior to our formal study, we took several hind limbs of normal rats to measure their knee joint ROM using this goniometer. We finally found that the knee joint can be pulled to approximately 140 degrees with a torque of 5.3N-cm. After that, an increase of torque can result in very small angle increase. Consequently, 5.3N-cm was used as a standardized torque to measure the knee joint ROM in our formal experiments.
8. ROM calculation: To ensure experimental accuracy, the ROM measurements were, respectively, carried out by two examiners, and each examiner repeated the measurements three times for each limb. The examiners were blinded to each other’s scores. Final value of each knee joint ROM was the mean of these six measurements taken by both examiners. Hence, a smaller flexion angle actually indicated a severe loss of motion and hinted the happening of extending knee joint contracture. The ROM of the left knee joint in each group was measured before and after myotomy with a standard torque of 5.3 N-cm.
9. Total and arthrogenic contracture calculation: The following formulas were used to calculate the degree of total and arthrogenic contracture: total contracture = ROM before myotomy (of the control knee)- ROM before myotomy (of the contracted knee); arthrogenic contracture = ROM after myotomy (of the control knee)—ROM after myotomy (of the contracted knee).

### Tissue preparation

The anterior joint capsules of rats were cut from the knee and used for following pathological and molecular examination after ROM measurements. Some samples were fixed with 4% paraformaldehyde overnight at 4℃, some samples were frozen in liquid nitrogen and then stored at − 80 °C, and some samples were fixed with fixed in 2.5% glutaraldehyde.

### Masson staining

Some samples used for Masson staining were fixed with 4% paraformaldehyde overnight at 4 ℃. The fixed joint capsule specimens were dehydrated in graded alcohol and then embedded in paraffin. The tissue paraffin block was sectioned at 5 μm and then deparaffinized. Masson staining was adopted to assess the level of collagen deposition. The Masson-stained sections were then observed under 200 × magnification, and six randomly selected fields were captured and analyzed to determine the percentage of the blue area which represented the degree of collagen deposition using Image ProPlus software (Media Cybernetics, Silver Spring, MD). The average percentage of the blue area from each slice was calculated and used as a measure of collagen deposition.

### Western blotting

The protein levels of HIF-1α, NLRP3, GSDMD-N, Caspase-1, TGF-β1, p-Smad3 and α-SMA were obtained using western blotting analysis. Total proteins were extracted from joint capsule specimens using RIPA lysis buffer with PMSF (Beyotime, China) on ice. BCA Protein Assay Kit (Beyotime, China) was used to detect the protein concentration. An equivalent amount of proteins of each pair specimens were separated by SDS-PAGE on 10% polyacrylamide gels and electrolytically transferred to polyvinylidene difluoride membranes (Millipore). The membranes were blocked in 5% skim milk dissolved in TBST (Tris-buffered saline with Tween-20) for 2 h at room temperature and then incubated with rabbit anti HIF-1α monoclonal antibody (1:1000; Wanlei), rabbit anti NLRP3 monoclonal antibody (1:2000; Bioss), rabbit anti GSDMD-N monoclonal antibody (1:1000; Abcam), rabbit anti Caspase-1 monoclonal antibody (1:1000; Affinity), rabbit anti TGF-β1 monoclonal antibody (1:1000; Bioss), rabbit anti p-Smad3 monoclonal antibody (1:1000; Bioss), rabbit anti α-SMA monoclonal antibody (1:500; Bioss) at 4℃ overnight. On the second day, after being washed in TBST solution three times for 10 min per wash, membranes were incubated with peroxidase-conjugated Affinipure goat anti-rabbit IgG-HRP (dilution 1:5000; Abcam, China) as the secondary antibody for 2 h at room temperature. After being washed three times with TBST for 10 min per wash, the membranes were then detected with the enhanced chemiluminescence system according to the manufacturer's instructions. The densities of bands were quantified using Image J software, and HIF-1α, NLRP3, GSDMD-N, Caspase-1, TGF-β1, p-Smad3 and α-SMA band densities were normalized with GAPDH.

### Transmission electron microscope

Joint capsule tissues were collected and fixed in 2.5% glutaraldehyde. After treated at 4 °C for 6–8 h, the samples were cut into 1-mm^3^-thick coronal slices. Next, the samples were rinsed with PBS (0.1 M) and injected with 1% osmic acid for 1-2 h. Subsequently, epoxy resin was used for embedding prior to slicing of the ultra-thin sections. Then, the copper mesh was retrieved, and the electron staining (lead staining) was carried out, and the photos were taken by a JEM-1400 transmission electron microscope (JEOL Ltd, Tokyo, Japan).

### Statistical analyses

All data in our study were presented as mean ± standard deviation and entered in SPSS Version 23.0. Different immobilization periods were considered as grouping variables in this study. The effects of different immobilization periods on the degree of knee contracture, pathology and molecular changes in joint capsule of rats were studied, so one-way analysis of variance was adopted. Levene's test was firstly used to evaluate regularity of variance. Bonferroni test was used for regular variance and Tamhane's T2 was used for irregular variance. A P-value of less than 0.05 was chosen as the significance threshold.

## Results

### Immobilization induced total and arthrogenic contracture in rats

Degrees of total and arthrogenic contracture are shown in Fig. 2B-C. Total and arthrogenic contracture of knee joints gradually increased with the prolongation of immobilization period. Degrees of total and arthrogenic contracture in the I-1 group were increased compared with the C group (*P* < 0.05). Degrees of total and arthrogenic contracture in the I-2 group were greater than those in the C and I-1 groups (*P* < 0.05). Furthermore, degrees of total and arthrogenic contracture of rats in the I-4 group were greater than those in the C, I-1 and I-2 groups (*P* < 0.05). Degrees of total and arthrogenic contracture in the I-6 group were higher than those in the C, I-1 and I-2 groups (*P* < 0.05). Degrees of total and arthrogenic contracture in the I-6 group were higher than those in the I-4 group but failed to reach a statistical difference (*P* > 0.05). Degrees of total and arthrogenic contracture in the I-8 group were higher than those in the C, I-1 and I-2 groups (*P* < 0.05). Degrees of total and arthrogenic contracture in the I-8 group were higher than those in the I-4 and I-6 groups but failed to reach a statistical difference (*P* > 0.05).

### Immobilization promoted collagen deposition in anterior joint capsule

**Fig. 3:**
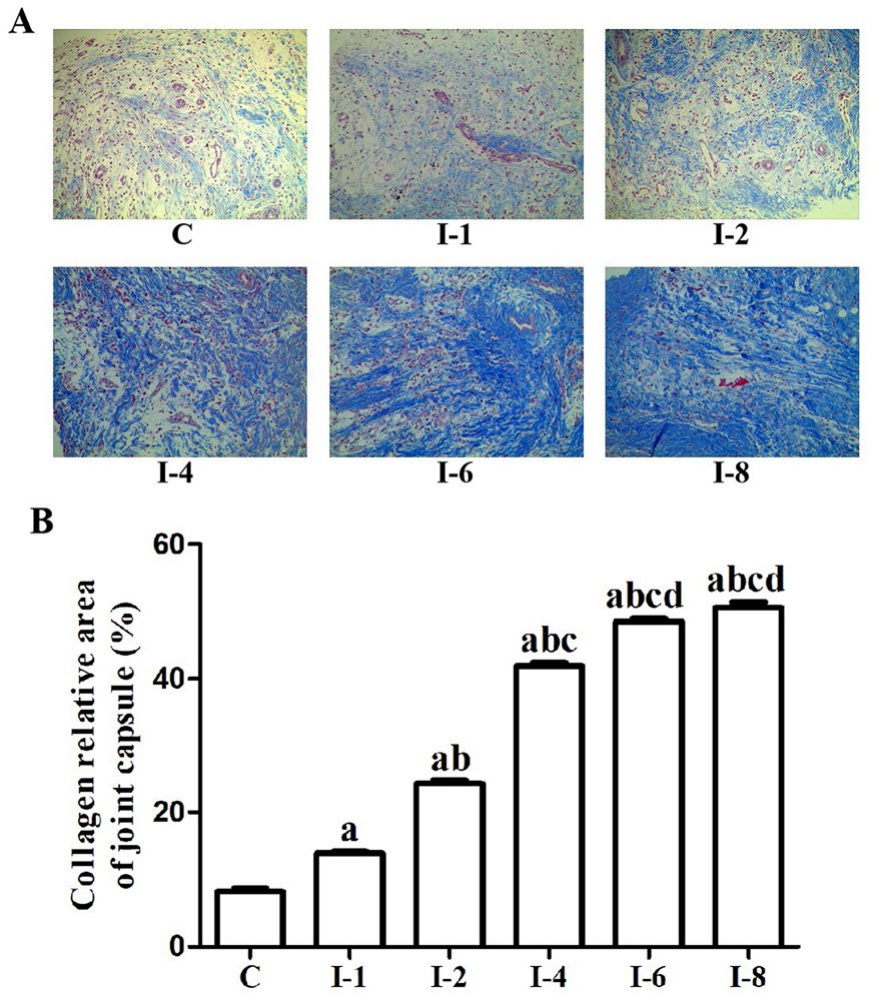
Immobilization promoted collagen deposition in anterior joint capsule. **A** Representative images of Masson staining. **B** Quantitative analysis of Masson staining. C, Rats that did not undergo immobilization; I-1, rats that underwent 1 week of immobilization; I-2, rats that underwent 2 weeks of immobilization; I-4, rats that underwent 4 weeks of immobilization; I-6, rats that underwent 6 weeks of immobilization; I-8, rats that underwent 8 weeks of immobilization

Masson staining indicated that immobilization can facilitate collagen deposition in anterior joint capsule (Fig. 3). Collagen deposition in anterior joint capsule was increased at 1 week after immobilization and remained elevated for 8 weeks. Statistical comparisons were made among different groups that underwent diverse periods of immobilization. After 1 week of immobilization, collagen deposition in anterior joint capsule in the I-1 group was statistically greater than that in the C group (*P* < 0.05). Moreover, collagen deposition in anterior joint capsule in the I-2 group was statistically greater than that in the C and I-1 groups (*P* < 0.05). Collagen deposition in anterior joint capsule in the I-4 group was statistically greater than that in the C, I-1 and I-2 groups (*P* < 0.05). Collagen deposition in anterior joint capsule in the I-6 group was statistically greater than that in the C, I-1, I-2 and I-4 groups (*P* < 0.05). Collagen deposition in anterior joint capsule in the I-8 group was statistically greater than that in the C, I-1, I-2 and I-4 groups (*P* < 0.05). Collagen deposition in anterior joint capsule in the I-8 group was greater than that in the I-6 group, but failed to reach a statistical difference (*P* > 0.05).

### Immobilization up-regulated the expression of HIF-1α in anterior joint capsule

**Fig. 4:**
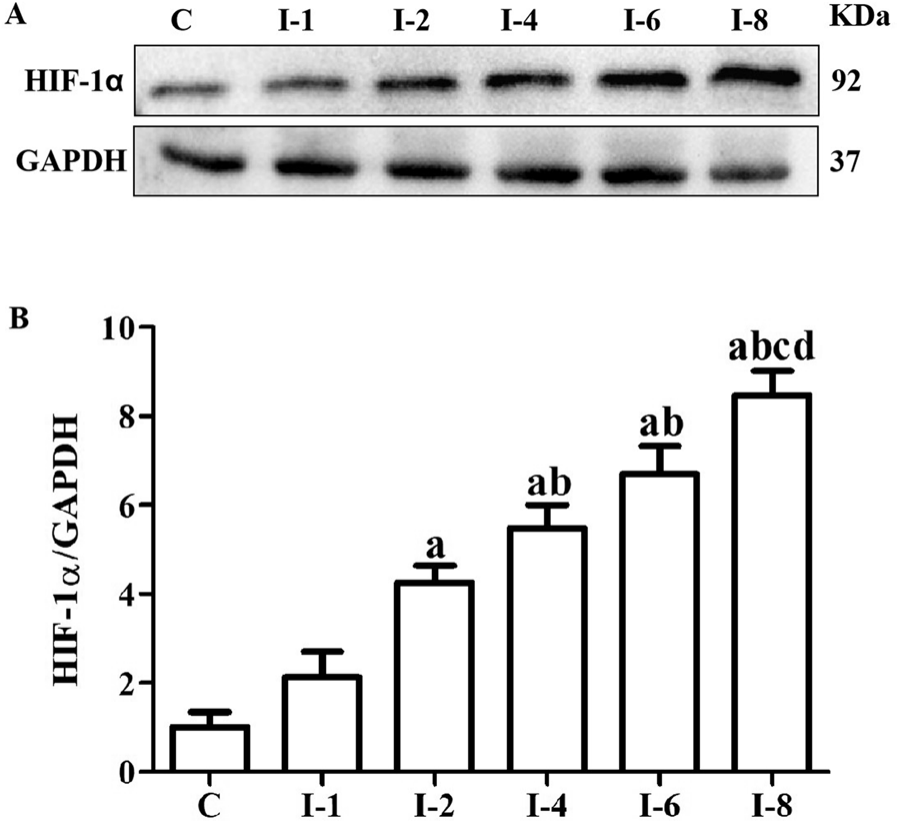
Immobilization up-regulated the expression of HIF-1α in anterior joint capsule. **A** Western blotting of HIF-1α. **B** Quantitative analysis of HIF-1α. C, Rats that did not undergo immobilization; I-1, rats that underwent 1 week of immobilization; I-2, rats that underwent 2 weeks of immobilization; I-4, rats that underwent 4 weeks of immobilization; I-6, rats that underwent 6 weeks of immobilization; I-8, rats that underwent 8 weeks of immobilization

Figure 4 illustrates the protein levels of HIF-1α relative to the average band intensity of GAPDH in the 6 groups. As shown in Fig. 4, protein expressions of HIF-1α increased following immobilization. There was no statistical difference between the C and I-1 groups (*P* > 0.05). The protein levels of HIF-1α in the I-2 group were statistically greater than those in the C group (*P* < 0.05). There was no statistical difference between the I-2 and I-1 groups (*P* > 0.05). The protein levels of HIF-1α in the I-4 group were statistically higher than those in the C and I-1 groups (*P* < 0.05). There was no statistical difference between the I-4 and I-2 groups (*P* > 0.05). The protein levels of HIF-1α in the I-6 group were statistically higher than those in the C and I-1 groups (*P* < 0.05). The protein levels of HIF-1α in the I-6 group were greater than those in the I-2 and I-4 groups, but failed to reach a statistical difference (*P* > 0.05). The protein levels of HIF-1α in the I-8 group were statistically higher than those in the C, I-1, I-2 and I-4 groups (*P* < 0.05). There was no statistical difference between the I-8 and I-8 groups (*P* > 0.05).

### Immobilization induced the activation of pyroptosis in anterior joint capsule

**Fig. 5:**
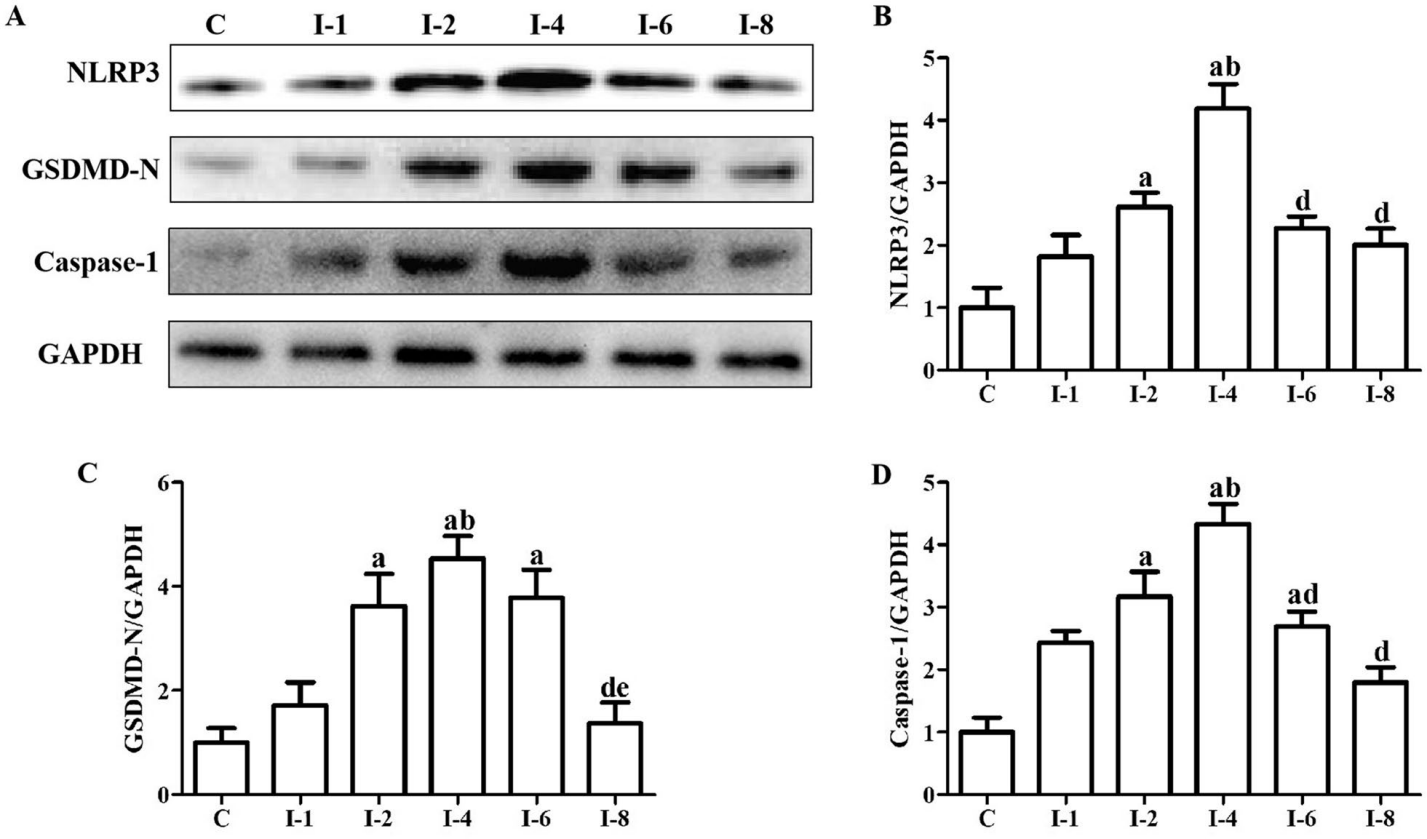
Immobilization induced the activation of pyroptosis in anterior joint capsule. **A** Western blotting of NLRP3, GSDMD-N and Caspase-1. **B** Quantitative analysis of NLRP3. **C** Quantitative analysis of GSDMD-N. **D** Quantitative analysis of Caspase-1. C,Rats that did not undergo immobilization; I-1, rats that underwent 1 week of immobilization; I-2, rats that underwent 2 weeks of immobilization; I-4, rats that underwent 4 weeks of immobilization; I-6, rats that underwent 6 weeks of immobilization; I-8, rats that underwent 8 weeks of immobilization

Figure 5 shows the activation condition of pyroptosis-related proteins. There was no statistical difference in the protein levels of NLRP3 in the C, I-1, I-6 and I-8 groups (*P* > 0.05). The protein levels of NLRP3 in the I-2 group were statistically greater than those in the C group (*P* < 0.05). There was no statistical difference in the protein levels of NLRP3 in the I-1 and I-2 groups (*P* > 0.05). The protein levels of NLRP3 in the I-4 group were statistically greater than those in the C and I-1groups (*P* < 0.05). The protein levels of NLRP3 in the I-2 and I-4 groups were not different (*P* > 0.05). The protein levels of NLRP3 in the I-6 group were lower than those in the I-2 group, but failed to reach a statistical difference (*P* > 0.05). The protein levels of NLRP3 in the I-6 group were statistically lower than those in the I-4 group (*P* < 0.05). The protein levels of NLRP3 in the I-8 group were lower than those in the I-2 group, but failed to reach a statistical difference (*P* > 0.05). The protein levels of NLRP3 in the I-8 group were statistically lower than those in the I-4 group (*P* < 0.05). There was no statistical difference in the protein levels of GSDMD-N in the C, I-1 and I-8 groups (*P* > 0.05). The protein levels of GSDMD-N in the I-2 group were statistically greater than those in the C group (*P* < 0.05). There was no statistical difference in the protein levels of GSDMD-N in the I-2 and I-1 groups (*P* > 0.05). The protein levels of GSDMD-N in the I-4 group were statistically greater than those in the C and I-1groups (*P* < 0.05). The protein levels of GSDMD-N in the I-4 group were higher than those in the I-2 group, but failed to reach a statistical difference (*P* < 0.05). The protein levels of GSDMD-N in the I-6 group were statistically greater than those in the C group (*P* < 0.05). The protein levels of GSDMD-N in the I-6 group were higher than those in the I-1 and I-2 groups and lower than those in the I-4 groups, but failed to reach a statistical difference (*P* > 0.05). The protein levels of GSDMD-N in the I-8 group were high than those in the C group and lower than those in the I-1 and I-2 groups, but failed to reach a statistical difference (*P* > 0.05). The protein levels of GSDMD-N in the I-8 group were statistically lower than those in the I-4 and I-6 groups (*P* < 0.05). There was no statistical difference in the protein levels of Caspase-1 in the C, I-1 and I-8 groups (*P* > 0.05). The protein levels of Caspase-1 in the I-2 group were statistically greater than those in the C group (*P* < 0.05). There was no statistical difference in the protein levels of Caspase-1 in the I-1 and I-2 groups (*P* > 0.05). The protein levels of Caspase-1 in the I-4 group were statistically greater than those in the C and I-1groups (*P* < 0.05). The protein levels of Caspase-1 in the I-2 and I-4 groups were not different (*P* > 0.05). The protein levels of Caspase-1 in the I-6 group were statistically greater than those in the C group and lower than those in the I-4 group (*P* < 0.05). The protein levels of Caspase-1 in the I-6 group were higher than those in the I-1 group and lower than those in the I-2 group, but failed to reach a statistical difference (*P* > 0.05). The protein levels of Caspase-1 in the I-8 group were high than those in the C group and lower than those in the I-1, I-2 and I-6 groups, but failed to reach a statistical difference (*P* > 0.05). The protein levels of Caspase-1 in the I-8 group were statistically lower than those in the I-4 group (*P* < 0.05).

### Immobilization induced the activation of TGF-β1/Smad3 signal pathway in anterior joint capsule

**Fig. 6:**
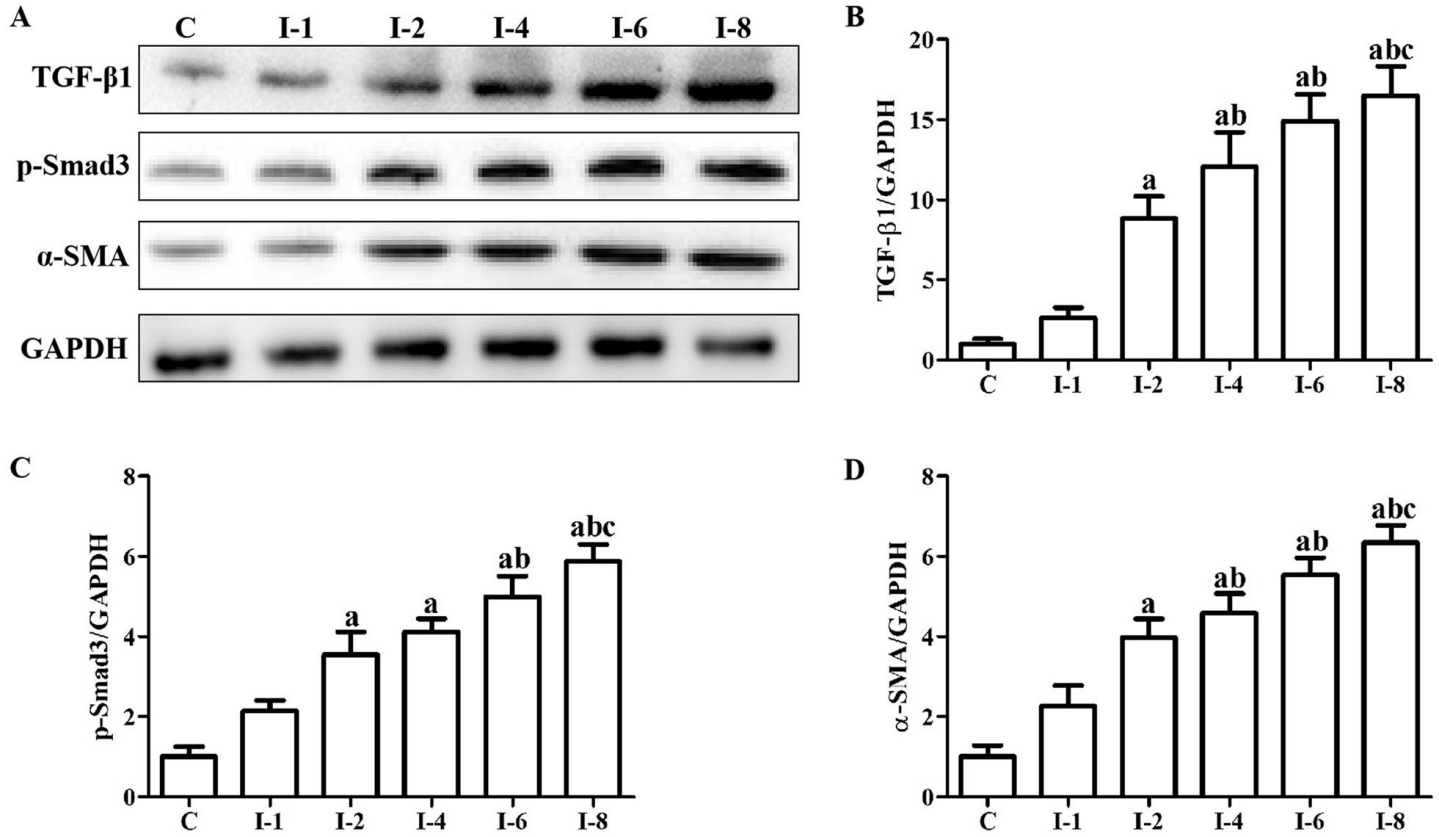
Immobilization induced the activation of TGF-β1/Smad3 signal pathway in anterior joint capsule. **A** Western blotting of TGF-β1, p-Smad3 and α-SMA. **B** Quantitative analysis of TGF-β1. **C** Quantitative analysis of p-Smad3. **D** Quantitative analysis of α-SMA. C, Rats that did not undergo immobilization; I-1, rats that underwent 1 week of immobilization; I-2, rats that underwent 2 weeks of immobilization; I-4, rats that underwent 4 weeks of immobilization; I-6, rats that underwent 6 weeks of immobilization; I-8, rats that underwent 8 weeks of immobilization

Figure 6 illustrates the activation condition of TGF-β/Smad signal pathway. There was no statistical difference in the protein levels of TGF-β1 in the C and I-1 groups (*P* > 0.05). The protein levels of TGF-β1 in the I-2 group were statistically greater than those in the C group (*P* < 0.05). There was no statistical difference in the protein levels of TGF-β1 in the I-1 and I-2 groups (*P* > 0.05). The protein levels of TGF-β1 in the I-4 group were statistically greater than those in the C and I-1 group (*P* < 0.05). There was no statistical difference in the protein levels of TGF-β1 in the I-2 and I-4 groups (*P* > 0.05). The protein levels of TGF-β1 in the I-6 group were statistically greater than those in the C and I-1 groups (*P* < 0.05). The protein levels of TGF-β1 in the I-6 group were greater than those in the I-2 and I-4 groups, but failed to reach a statistical difference (*P* > 0.05). The protein levels of TGF-β1 in the I-8 group were statistically greater than those in the C, I-1 and I-2 group (*P* < 0.05). The protein levels of TGF-β1 in the I-8 group were greater than those in the I-4 and I-6 groups, but failed to reach a statistical difference (*P* > 0.05). There was no statistical difference in the protein levels of p-Smad3 in the C and I-1 groups (*P* > 0.05). The protein levels of p-Smad3 in the I-2 group were statistically greater than those in the C group (*P* < 0.05). There was no statistical difference in the protein levels of p-Smad3 in the I-1 and I-2 groups (*P* > 0.05). The protein levels of p-Smad3 in the I-4 group were statistically greater than those in the C group (*P* < 0.05). The protein levels of p-Smad3 in the I-4 group were higher than those in the I-1 and I-2 groups, but failed to reach a statistical difference (*P* > 0.05). The protein levels of p-Smad3 in the I-6 group were statistically greater than those in the C and I-1 group (*P* < 0.05). The protein levels of p-Smad3 in the I-6 group were greater than those in the I-2 and I-4 groups, but failed to reach a statistical difference (*P* > 0.05). The protein levels of p-Smad3 in the I-8 group were statistically greater than those in the C, I-1 and I-2 group (*P* < 0.05). The protein levels of p-Smad3 in the I-8 group were greater than those in the I-4 and I-6 groups, but failed to reach a statistical difference (*P* > 0.05). There was no statistical difference in the protein levels of α-SMA in the C and I-1 groups (*P* > 0.05). The protein levels of α-SMA in the I-2 group were statistically greater than those in the C group (*P* < 0.05). There was no statistical difference in the I-1 and I-2 groups (*P* > 0.05). The protein levels of α-SMA in the I-4 group were statistically greater than those in the C and I-1 group (*P* < 0.05). There was no statistical difference in the protein levels of α-SMA in the I-2 and I-4 groups (*P* > 0.05). The protein levels of α-SMA in the I-6 group were statistically greater than those in the C and I-1 groups (*P* < 0.05). The protein levels of α-SMA in the I-6 group were greater than those in the I-2 and I-4 groups, but failed to reach a statistical difference (*P* > 0.05). The protein levels of α-SMA in the I-8 group were statistically greater than those in the C, I-1 and I-2 group (*P* < 0.05). The protein levels of α-SMA in the I-8 group were greater than those in the I-4 and I-6 groups, but failed to reach a statistical difference (*P* > 0.05).

### Immobilization induced pyrosis of fibroblast in joint capsule

**Fig. 7:**
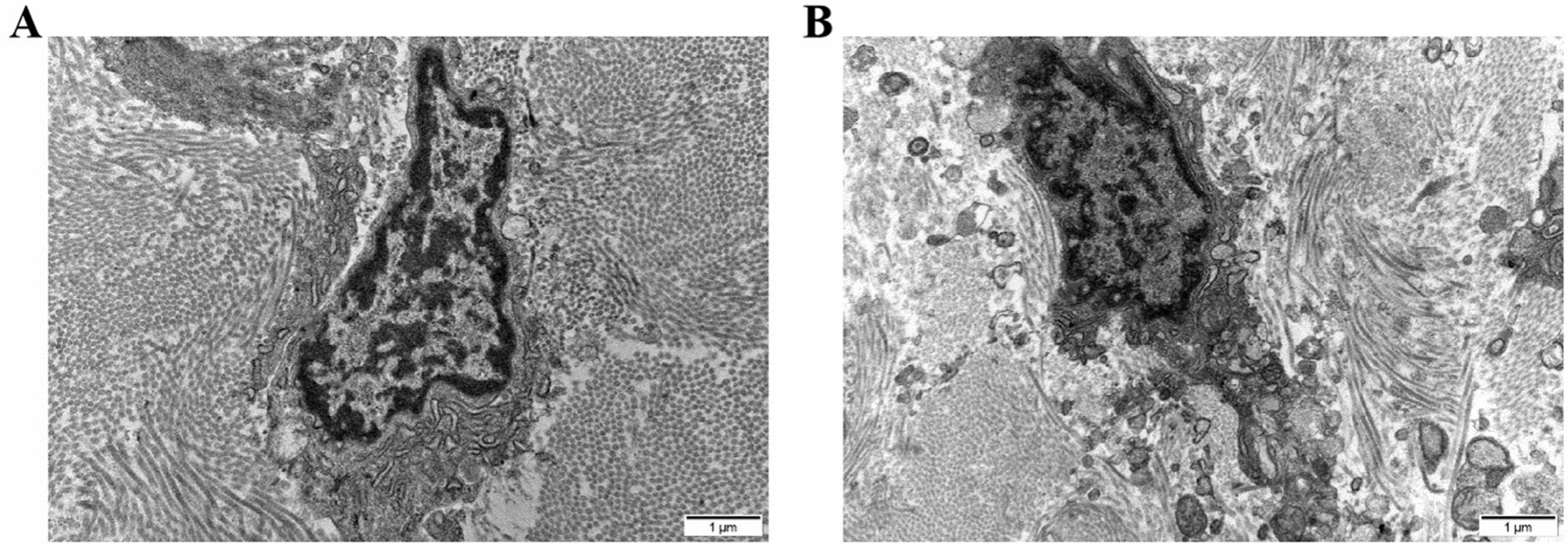
Immobilization induced pyroptosis of fibroblast in joint capsule. **A** Representative image of transmission electron microscope in normal rats. **B** Representative image of transmission electron microscope in rats immobilized for four weeks

The results of transmission electron microscope are shown in Fig. 7. In the normal control group, there was no cell membrane rupture and the nucleus was intact. Nevertheless, the cell membrane of the rat joint capsule fibroblast was broken and there was a spillover of the cell contents following 4 weeks of immobilization.

## Discussion

Joint contracture is a common complication of many musculoskeletal and neural disorders that can result in disabilities in patients. Joint immobilization is an effective treatment for the injury of the tissues around joints, but prolonged or inappropriate immobilization is also one of the common predisposing factors inducing joint contracture. Many molecular or morphological changes in the periarticular tissues may happen after the development of joint contracture. Joint capsule fibrosis is identified as the most significant morphological changes after the formation of joint contracture [28, 29]. TGF-β/Smad signal pathway is deemed to be the final signal pathway resulting in joint capsule fibrosis. However, the exact mechanism that can play a regulatory role on TGF-β/Smad signal pathway in immobilization induced joint capsule fibrosis is still rarely known. Pyroptosis have been reported to play a promoting role in fibrosis of many fibrotic diseases [30–33]. However, there was no study on the role of pyroptosis in the pathogenesis of joint contracture. In order to further investigate the regulatory role of pyroptosis in the development of joint capsule fibrosis and joint contracture, we designed the study.

Establishment of an ideal animal model is of great importance to the investigation on disease process. In order to observe the natural process of joint contracture, we established an animal model using immobilization by aluminum plates. Joint contracture is characterized by a reduction of joint range of motion compared with normal joints [34, 35], thus decreased join range of motion represents increased degree of joint contracture. Previous studies have suggested that joint contracture occur within 1 week of knee joint immobilization and progress in a time-dependent manner [36, 37]. Nagai M et al. [36] reported that myogenic factors play a major role in limiting joint ranges of motion within 2 weeks of immobilization, and arthrogenic factors play a major role in limiting joint ranges of motion thereafter. Moreover, Chimoto et al. [38] found that 4 weeks of knee joint immobilization can result in stable joint contracture, 6 weeks of knee joint immobilization can cause severe joint contracture, and the degree of joint contracture became plateau after 8 weeks of immobilization. In order to observe the effect of different periods of immobilization on joint contracture in this animal model and explore the underlying mechanism, the rats in different groups were immobilized for 1, 2, 4, 6 or 8 weeks. Biomechanical findings in this study showed greater joint ranges of motion loss after different periods of immobilization, which indicated that animal model of joint contracture is successfully established. Furthermore, the study indicated that joint ranges of motion loss progress rapidly in the first 4 weeks and then progress slowly, which illustrated that interventions need to be made in the early stage of joint contracture.

Relatively, the results of histological findings in our study were consistent with the biomechanical studies. Collagen deposition is deemed to be the most important pathological change that can directly cause tissue fibrosis [39, 40]. Collagen deposition gradually increased until the first 8 weeks of immobilization, which indicated joint capsule fibrosis happened with the progression of joint contracture. Previous studies have shown that TGF-β/Smad signal pathway play an important pole in the progression of joint capsule fibrosis and joint contracture [30, 41]. Fibroblasts and myofibroblasts are important effector cells in the pathogenesis of joint capsule fibrosis in the development of joint contracture [4]. During pathological fibrosis progression, fibroblasts are highly activated, which can then result in excessive ECM production and collagen deposition [42]. It has been widely reported that transforming growth factor-β1 (TGF-β1) is a pro-fibrotic factor that can up-regulate the function of fibroblasts through the TGF-β/Smad pathway and then result in tissue fibrosis [43]. Previous studies have demonstrated that TGF-β1 can induce phosphorylation of the downstream Smad proteins, predominantly Smad3, leading to collagen production [44]. In our study, results of western blotting revealed that protein levels of TGF-β1 increased continually, along with the increased protein expression of α-SMA and p-Smad3, which indicated the activation of TGF-β1/Smad3 signal pathway. These findings seem to be in accordance with previous studies concerning joint contracture using other animal models [2, 5].

Tissue activities reduced and hypoxia may be induced following immobilization. Our previous studies have shown that unilateral lower limb immobilization can result in the elevated expression of HIF-1α in rabbits rectus femoris, which hinted hypoxia happened following immobilization [45, 46]. In this study, the protein levels of HIF-1α gradually increased with the prolongation of immobilization, which implied hypoxia existed persistently during the first 8 weeks of immobilization. NLRP3 is a well-known inflammasome that can sense plasma membrane perturbations caused by certain microbial products or sterile danger signals and then lead to cell death, such as pyroptosis [47]. Moreover, studies have indicated that HIF-1α can promote the expression of NLRP3 under hypoxia environment [48, 49]. Consistent with the elevated expression of HIF-1α, protein levels of NLRP3 also increased with the prolongation of immobilization. Thus, we guessed that NLRP3 expression can be up-regulated via HIF-1α in anterior joint capsule under the environment of hypoxia following immobilization.

As a pyroptosis inducer, NLRP3 is known to have an promoting effect of pyroptosis. It was reported that caspase-1 was activated after the induction of NLRP3, and gasdermin D (GSDMD) was cleaved, and the N-terminal domain can oligomerize to form pores in the cell membrane, inducing pyroptosis [50]. In our study, immobilization markedly increased the protein levels of caspase-1 and GSDMD-N, which indicated the activation of pyroptosis. Additionally, results of transmission electron microscope in our experiment indicated that cell membrane of the rat joint capsule fibroblast was broken, there was a spillover of the cell contents, and the nucleus was pasty after 4 weeks of immobilization, which further hinted the activation of pyroptosis. In general, the results indicated that hypoxia may facilitate joint capsule fibrosis via the activation of pyroptosis.

## Conclusion

To our knowledge, this is the first study to elucidate that hypoxia-mediated pyroptosis may play a role in the development of joint capsule fibrosis and joint contracture. Improving joint capsule hypoxia and then reversing pyroptosis may be an effective method for the treatment of joint contracture, and this may be one of our future research priorities.

## Data Availability

Data are available from the first author on reasonable request.
