# Correction: Role of hypoxia-mediated pyroptosis in the development of extending knee joint contracture in rats

**DOI:** 10.1186/s40001-024-01960-y

**Published:** 2024-07-15

**Authors:** Quan-Bing Zhang, Lei Huo, Mian Li, Rui Zhang, Ting Zhou, Feng Wang, Yun Zhou

**Affiliations:** 1 Department of Rehabilitation Medicine, Economic and Technological Development Zone, The Second Affiliated Hospital of Anhui Medical University, No. 678 Furong Road, Hefei, 230601 Anhui China; 2 Hefei Institute of Physical Sciences, Chinese Academy of Sciences, Hefei, 230031 Anhui China

**Correction: European Journal of Medical Research (2024) 29:298** 10.1186/s40001-024-01890-9

Following publication of the original article [1], the author would like to correct the figure caption of Fig. 7 from “Fig. 7 Immobilization induced pyroptosis of fibroblast in joint capsule. A Representative images of transmission electron microscope in normal rats. B Representative images of transmission electron microscope in rats immobilized for four weeks. **C Rats that did not undergo immobilization; I-1, rats that underwent 1 week of immobilization; I-2, rats that underwent 2 weeks of immobilization; I-4, rats that underwent 4 weeks of immobilization; I-6, rats that underwent 6 weeks of immobilization; I-8, rats that underwent 8 weeks of immobilization”** to “Fig. 7 Immobilization induced pyroptosis of fibroblast in joint capsule. A Representative images of transmission electron microscope in normal rats. B Representative images of transmission electron microscope in rats immobilized for four weeks.”

**Fig. 7:**
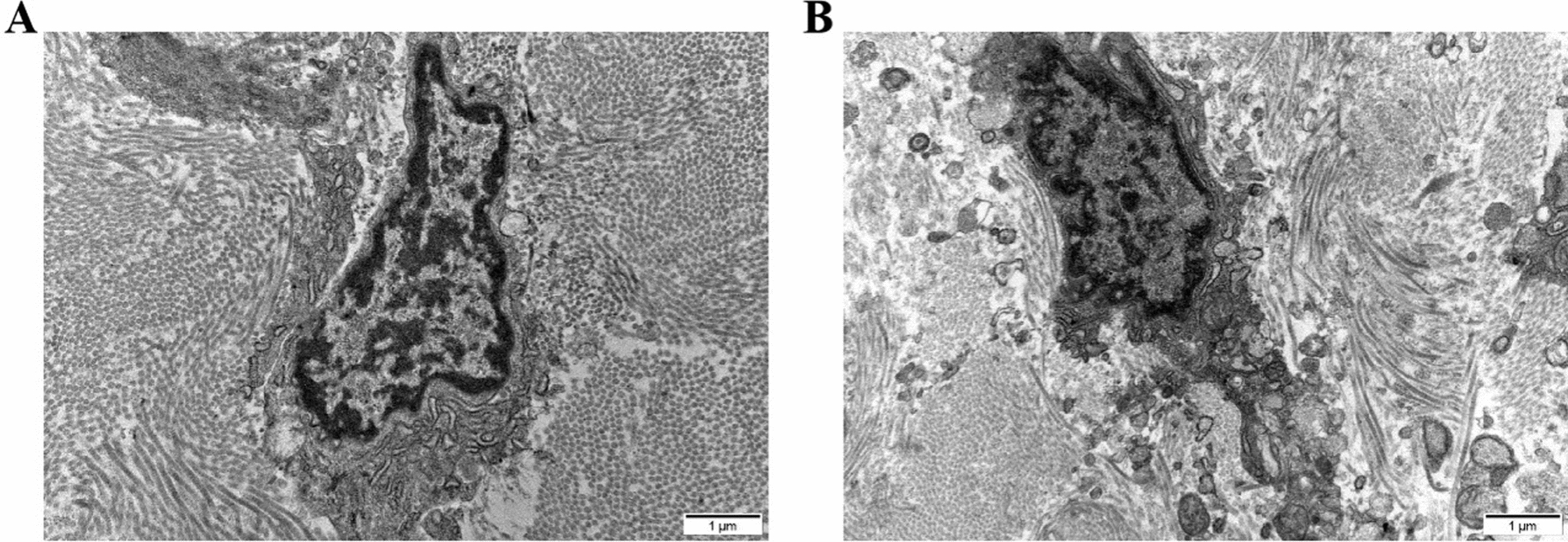
Immobilization induced pyroptosis of fibroblast in joint capsule. **A** Representative images of transmission electron microscope in normal rats. **B** Representative images of transmission electron microscope in rats immobilized for four weeks

The bold interface needs to be removed in this correction and the original article has been corrected.
